# Molecular cloning and expression analysis of the *aqp1aa* gene in half-smooth tongue sole (*Cynoglossus semilaevis*)

**DOI:** 10.1371/journal.pone.0175033

**Published:** 2017-04-05

**Authors:** Hua Guo, Min Wei, Yang Liu, Ying Zhu, Wenteng Xu, Liang Meng, Na Wang, Changwei Shao, Sheng Lu, Fengtao Gao, Zhongkai Cui, Zhanfei Wei, Fazhen Zhao, Songlin Chen

**Affiliations:** 1 Yellow Sea Fisheries Research Institute, Chinese Academy of Fishery Sciences (CAFS), Key Laboratory for Sustainable Development of Marine Fisheries, Ministry of Agriculture, Qingdao, PR China; 2 College of Fisheries and Life, Shanghai Ocean University, Shanghai, PR China; 3 Wuxi Fisheries College, Nanjing Agricultural University, Wuxi, PR China; 4 Laboratory for Marine Fisheries Science and Food Production Processes, Qingdao National Laboratory for Marine Science and Technology, Qingdao, China; Xiamen University, CHINA

## Abstract

Aquaporin 1 (AQP1) is a member of the transmembrane water channel family of proteins with special structural features, and two AQP1 paralogous genes (*aqp1aa* and *aqp1ab*) are reported in teleosts. In the present study, the *aqp1aa* gene of half-smooth tongue sole (*Cynoglossus semilaevis*) was cloned and characterized. The full-length cDNA of *aqp1aa* is 1411 bp with a 786 bp open reading frame encoding a 261-amino acid putative protein with a characteristic structure consisting of 6 membrane-spanning α-helical domains and two highly conserved asparagine—proline—alanine motifs. Real-time quantitative PCR revealed that *aqp1aa* mRNA is expressed predominantly in the testis of males and pseudo-males, while its expression is low in the ovary and lowest in doublesex and mab-3-related transcription factor 1(*DMRT1*) knock out fish and triploid males. *In situ* hybridization indicated that *aqp1aa* mRNA is expressed mainly in the germ cells of males and pseudo-males, especially in spermatozoa and spermatids. These results suggest that the *aqp1aa* may play a role in spermatogenesis of *C*. *semilaevis*.

## Introduction

Half-smooth tongue sole (*Cynoglossus semilaevis*) is an economically important marine fish that is widely distributed along the Chinese coast. It is widely cultivated in the north of China because of its delicious taste and nutrition. *C*. *semilaevis* presents the ZW type sex-determination system characterized by heterogametic ZW females and homogametic ZZ males [1]. In addition, some genetically female individuals transform into phenotypically male individuals by sex reversal, which are then named pseudo-males with the male heterogametic ZW [2]. Pseudo-males are fertile and can mate with normal females to produce viable offspring, which demonstrate that pseudo-males can produce normal spermatozoa same as ZZ males [1]. However, artificial ZZZ triploid males of tongue sole cannot produce normal sperm due to the inhibition of testis development [3–4]. Furthermore, *DMRT1* is a vital gene that is involved in the male sex-determination of tongue sole [2, 5], and the spermatogenesis process was disrupted in the ZZ dmrt1 mutant tongue sole. Compared to wild-type males, only a few spermatogonia, and few or in some cases even no spermatocytes and spermatids were observed [5]. Although the whole genomic sequence of *C*. *semilaevis* has been sequenced [2] and several spermatogenesis related genes (such as *tesk1*, *piwil2*, *Figla_tv2*, *Patched1*, and *neurl3*) involved in gonadal development have been studied [6–10], the mechanisms of spermatogenesis in half-smooth tongue sole remain unclear.

Spermatogenesis is a major male reproductive event, in which mature spermatozoa produced in testis. The germ line stem cells embedded in Sertoli cells divide into spermatogonia by cell mitosis. After two meiotic divisions of spermatogonia, the haploid spermatids are produced. Subsequently, the spermatids undergo a molecular and morphological remodeling process including differentiation of the head, middle piece, and flagellum of the spermatozoa [11]. Maintaining fluid homeostasis during spermatogenesis and sperm maturation is vital for male fertility in the mammalian and teleost testis [12–13], and osmotic changes associated with water and ion fluxes are also critical for the activation of sperm motility and subsequent natural fertilization [14–16]. Therefore, the potential role of transmembrane water channel proteins such as aquaporins has received particular consideration due to the significance of fluid transport and efficient cell volume regulation during spermatogenesis and sperm motility.

Aquaporins (AQPs) are transmembrane water channel proteins that allow water and some small molecules to pass through the plasma membrane of cells in animals [17]. To date, a diversity of the aquaporin gene superfamily have been discovered in prokaryotic and eukaryotic organisms [18–29]. 17 classes of aquaporins (Aqp0—Aqp16) have been reported in the eukaryotic organisms and segregated into four major grades including classical aquaporins (Aqp0, 1, 2, 4, 5, 5L, 6, 14 and 15), Aqp8-type of aquaammoniaporins (Aqp8 and 16), aquaglyceroporins (Aqp3, 7, 9, 10 and 13), and unorthodox aquaporins (Aqp11 and 12) [19]. Aquaporin 1 (AQP1) is the first isoform identified on the cell membrane of erythrocytes [30], its monomers contain water pores but associate in the membrane as tetramers, each monomer typically contains six transmembrane α-helices with the N- and C-termini both located on the cytoplasmic side of the membrane [31–32], and its role in cells has been extensively studied and well understood as a water-selective channel. However, subsequent studies have revealed that AQP1 has other roles in living organisms, including cell migration, spermatogenesis and neural signal transduction [17, 33–34]. In mammals, AQP1 has been recognized in the testis and is involved in the early stage of spermatogenesis [35–36], where it may play a role in the secretion of tubule liquid to achieve the luminal changes required for sperm concentration transition and maturation [12, 37]. In marine teleosts, two AQP1 paralogous genes (*aqp1aa* and *aqp1ab*) are reported [20, 38–45]. The *aqp1ab* predominately expressed in ovary and involved in the hydration of the oocyte [40–41, 46–48], while *aqp1aa* mainly expressed in spermatozoa, especially located flagellum of the spermatozoa in marine teleost (*Sparus aurata*) [38]. Besides, the *aqp1aa* is involved in the sperm motility activation by mediating the water efflux during hyperosmotic shock [38–39, 43, 45, 49]. Although the cloning of *aqp1aa* gene in tongue sole has been published [40], its function in tongue sole is still unclear.

In the present study, we obtained the full-length cDNA of *aqp1aa*in *C*. *semilaevis* and analyzed the sequence characteristics. In addition, the relative expression of *aqp1aa* in different tissues and stages of gonadal development was assessed. To analyze the expression differences among different germ cells, *in situ* hybridization (ISH) was also conducted. The results indicate that *aqp1aa* is likely to play a role in spermatogenesis of tongue sole.

## Materials and methods

### Ethics statement

All experiments involving *C*. *semilaevis* in this study have been approved by the Yellow Sea Fisheries Research Institute’s animal care and use committee. Great efforts have been made to minimize fish suffering.

### Experimental animals and sample collection

The *C*. *semilaevis* used in the experiments were purchased from the Haiyang High-Tech Experimental Base (Haiyang, China), and genetic and phenotypic sexuality was determined by an established method [50]. The fish were randomly selected (three individuals of each gender), and tissues from 2 years post-hatching (yph) fish (spleen, heart, intestine, brain, kidney, liver, gill, gonad, and gamete) were collected and snap-frozen in liquid nitrogen. The gonads of fish at different development stages (34, 52, 80, 105 and 210 days, 1 and 2 years) were obtained for RNA preparation. The gonadal cell lines produced in our laboratory were established from the gonads of males and females (500 g and 250 g, respectively) [51–52], and they were thawed from cryopreservation and collected for RNA extraction at passage 12 (S1 Fig). The gonads of different genotypes were collected from males, females, pseudo-males, (ZZZ) triploid males, and (ZZ) *DMRT1*-knock out fish (all fish were 2 yph). Triploid males were produced using hydraulic pressure shock, and triploidy was validated by karyotyping and flow cytometry analysis [3]. *DMRT1*-knock out fish was also produced in our laboratory by microinjecting the TALEN mRNAs targeted towards dmrt1 into sole embryos (wild-type) [5]. Total RNA was extracted with a TRIzol isolation kit (Qiagen, Dusseldorf, Germany). For the *in situ* hybridization, the fresh gonads (210 dph, 1 and 2 yph) were fixed in 4% paraformaldehyde (PFA) overnight at 4°C and stored in 70% ethyl alcohol at -20°C.

### RACE PCR

To carry out a rapid amplification of the cDNA ends (5’ and 3’ RACE), two pairs of specific primers (S1 Table) were designed based on the partial cDNA sequence from half-smooth tongue sole genome sequencing. To obtain the full-length cDNA of *aqp1aa*, the 5’ and 3’ RACE PCR was conducted using the SMART RACE cDNA Amplification Kit (Clontech Inc., Mountain View, CA, USA). The universal primer (UPM) and outer primers were used for the 5’ and 3’ RACE outer amplifications; then, the outer PCR amplification products were diluted 50 times with ddH_2_O and used as a template for the inner PCR amplifications with the universal primer (NUP) and the inner primers. The program of touchdown PCR was performed as follow: five cycles (94°C for 30 s and 72°C 3 min); five cycles (94°C for 30 s, 70°C 30 s, and 72°C for 3 min); five cycles (94°C for 30 s, 68°C 30 s, and 72°C for 3 min); twenty cycles (94°C for 30 s, 66°C 30 s, and 72°C for 3 min); and then 72°C for 7 min for elongation. The PCR products were electrophoresed on a 1.5% agarose gel, and the band of the expected size was cut and purified with the Universal DNA Purification Kit (Tiangen, Beijing, China). The expected purified fragments were cloned into the pEasyT1 vector from Transgen Biotech (Transgen, Beijing, China), propagated in Escherichia Coli transT1 (Transgen, Beijing, China), and positive clones were selected and sequenced by the Ruibiotech Company (Beijing, China).

### Bioinformatics sequence analysis

The sequence data were analyzed using the DNAStar 7.0 software (DNAStar, Madison, WI, USA). The predicted protein sequence was obtained using the ExPASy translation tools (http://www.expasy.ch/). The conserved domains or motifs were analyzed using the NCBI conserved domain search (http://www.ncbi.nlm.nih.gov/structure). The physicochemical characteristics of the protein sequence were computed by ProtParam (http://www.expasy.org/tools/protparam/). The protein structure and ligand-binding site were predicted with the Raptorx program (http://raptorx.uchicago.edu/) [53]. The transmembrane helices of the protein were predicted by the TMHMM Server v. 2.0 software (http://www.cbs.dtu.dk/services/TMHMM/).

### Phylogenetic analysis

The phylogenetic analysis was performed using the Bayesian inference (BI) analysis. The nucleotide sequences of *AQP1* from *C*. *semilaevis* and other vertebrates were downloaded from the GenBank database (http://www.ncbi.nlm.nih.gov/) and aligned using the ClustalW method (MEGA v6.0) and then filtered to remove ambiguous positions. The best model was selected with the PartitionFinder v1.1.1 software [54] with the “greedy” algorithm, with branch lengths estimated as “linked” and applying the Bayesian information criterion (BIC). The BI analyses were conducted with the MrBayes v3.2 software [55] and run for one million generations with trees sampled every 100 generations. The first 25% of the trees was burned. The remaining trees were used for constructing the Bayesian consensus tree. Finally, the tree was saved and edited with the FigTree v1.4.2 software (http://tree.bio.ed.ac.uk/software/figtree/).

### Real-time quantitative PCR

First strand cDNA synthesis was conducted using a PrimeScript RT regent Kit with gDNA Eraser (TAKARA, Dalian, China). Quantitative PCR (qPCR) was conducted on a 7500Fast Real time PCR system (Applied Biosystems, USA) in a 20 μl reaction, containing 1 μl cDNA template, 10 μl SYBR Premix Ex Taq^™^ (Takara), 0.4 μl ROX reference dye II, 0.4 μl of AQP1-RT-F and AQP1-RT-R (Actin-F and Actin-R for the reference gene, S1 Table). The primer of qPCR was designed to cross the boundary of intron and exon (S2 Fig). The amplification procedure of the qPCR was as follows: 95°C for 30 s, followed by 40 cycles at 95°C for 5 s and then 60°C for 34 s. Disassociation curve analysis was conducted to determine target specificity. *β-actin*, a suitable reference gene in tongue sole (S2 Fig), was used as the internal reference [56]. Each sample was analyzed in triplicate and at least three samples at the same stage were processed. The relative expression of mRNA was calculated using the 2^–ΔΔCt^ method [57]. The differences between groups were analyzed using one-way ANOVA with the SPSS 18.0 software (IBM, New York, NY, USA). The significance level of data variance was set at 0.05.

### *In situ* RNA hybridization

The specific primers (AQP1-ISH-F and AQP1-ISH-R) were designed in *aqp1aa* ORF domain (S1 Table), and the higher difference region was chosen as target region to amplify the ISH probe for avoiding cross-hybridization with *aqp1ab* (S3 Fig). The purified target fragment was inserted into the pGEM-T vector after identification by sequencing. The selected positive plasmid with a different direction of insertion was linearized with *NotI* and then used as a template for in vitro transcription with the T7 RNA polymerase. DIG-NTP was used to label RNA probes. The procedure of *in situ* hybridization was performed as previous described [2, 6], and three samples for each gender were processed.

## Results

### Cloning and characterization of *aqp1aa*

The full-length cDNA of *aqp1aa* was 1411 bp (GenBank accession number: KX904930), and contained a 131 bp 5’ untranslated region (UTR), a 786 bp open reading frame (ORF), and a 494 bp 3′ UTR with a single typical polyadenylation signal (AATAAA) at position 1,355, just 19 bp upstream from the poly (A) tail (S4 Fig). To illustrate the splicing of *aqp1aa*, the ORF sequence was mapped on the genomic sequence (GenBank accession number: NC_024326). The *aqp1aa* contained 4 exons (1–4) separated by 3 introns (A-C) (S5 Fig). It encoded a putative protein with 261 amino acid residues with a 27.48 kDa predicted molecular weight and 5.77 isoelectric point (PI). Through conserved domain searching and the PROSITE patterns searching, an amphipathic channel was detected (S4 Fig). The protein consists of six transmembrane (TM) domains with the highly-conserved asparagine-proline-alanine (NPA) boxes (S6 Fig), which are the hallmark of the MIP family of proteins to which AQPs belong.

### Phylogenetic analysis

**Fig 1:**
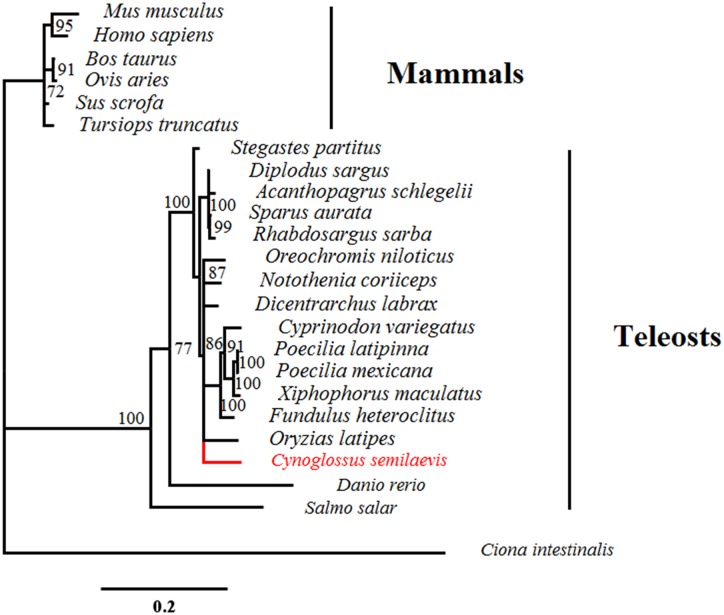
Phylogenetic tree of *aqp1aa* in 24 species using the Bayesian Inference (BI) method. *Ciona intestinalis* was chosen as out-group. Numbers at nodes represent BI posterior probabilities (percent).

To evaluate the evolutionary relationship of *aqp1aa* in different species, we conducted a phylogenetic analysis based on the nucleotide sequences *of aqp1aa* from 24 species using the BI analysis. In addition to that of *C*. *semilaevis*, 23 nucleotide sequences from 23 species, including 6 species of mammals, 17 species of teleosts, and 1 species of invertebrates (used as out-group), were downloaded from the GenBank database (S2 Table). The Bayesian posterior probability (PP) method was used to evaluate the clade support. As shown in Fig 1, the sequences mainly clustered in the following two groups: teleosts (pp = 1) and mammals (pp = 0.72). In the teleosts cluster, *C*. *semilaevis* forms a clade together with *Oreochromis niloticus*, *Notothenia coriiceps*, *Dicentrarchus labrax*, and *Oryzias latipes* (pp = 0.86).

### The expression pattern of *aqp1aa*

**Fig 2:**
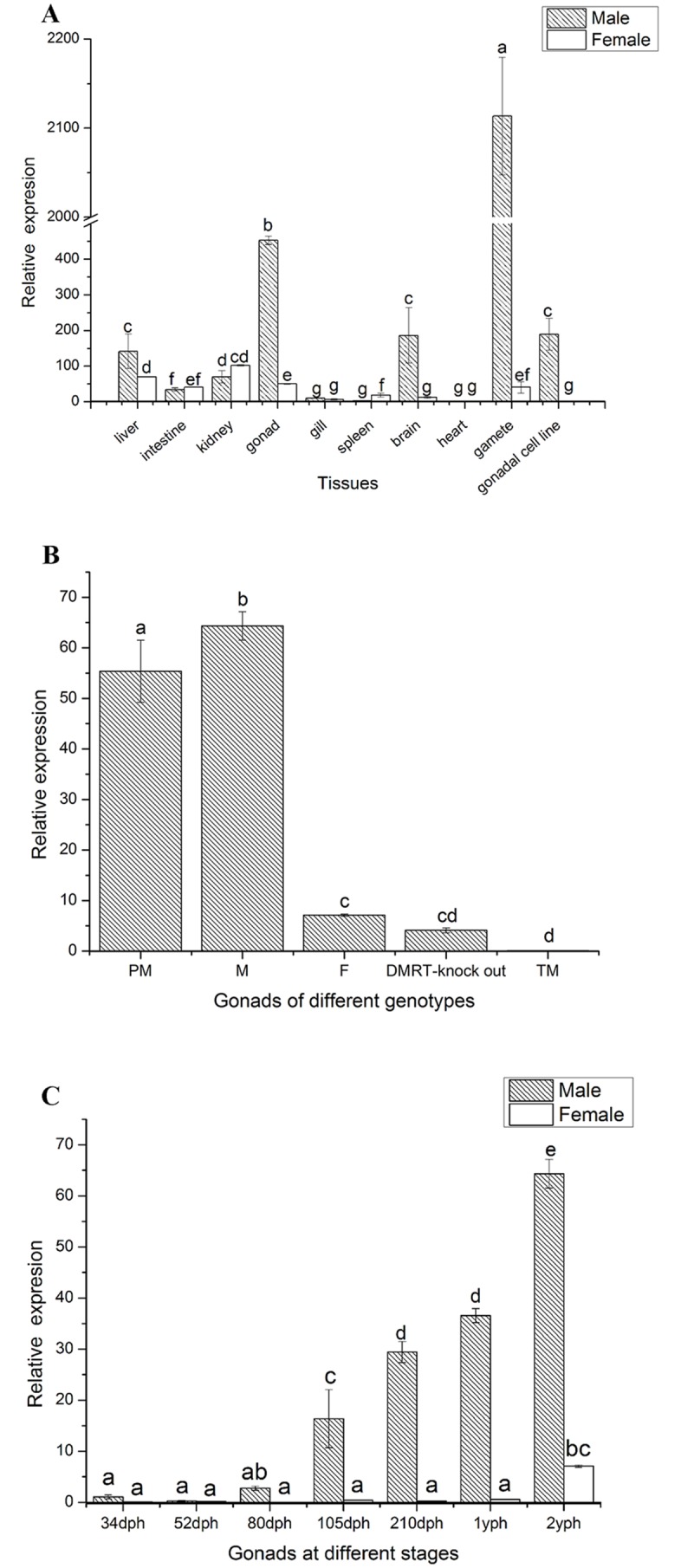
Quantitative PCR analysis of *aqp1aa* in *C*. *semilaevis*. (**A**) The expression of *aqp1aa* in various tissues of tongue sole. (**B**) The expression of *aqp1aa* in gonads of different genotypes. PM: ZW pseudo-male, M: ZZ male, F: ZW female, *DMRT1*-knock out: ZZ *DMRT1*-knock out fish, TM: ZZZ triploid male. (**C**) The expression of *aqp1aa* at different developmental stages of the gonads. The *aqp1aa* mRNA amount was normalized using *β*-actin. The data were analyzed by one-way ANOVA followed by Duncan comparison tests using the SPSS 18.0 software. Bars represent the triplicate mean ± SEM from three separate individuals (n = 3). Bars with different letters denote statistically significant differences (*P* < 0.05).

To evaluate the expression levels of *aqp1aa* in different tissues of 2 yph tongue sole, its mRNA was quantified in a wide range of tissues including spleen, heart, intestine, brain, kidney, liver, gill, gonads (testis and ovary), gametes (spermatozoa and eggs), and gonadal cell lines. As shown in Fig 2A, the highest mRNA expression was observed in the spermatozoa and testis (*P*<0.05), where *aqp1aa* expression was more than 9 times higher than that of eggs and ovary, respectively. High mRNA expression was also observed in a testicular cell line (*P*<0.05), while no mRNA expression was detected in the ovarian cell line. Regarding the other tissues, low levels of expression were detected in liver, kidney, intestine and male brain, while the expression levels of remaining tissues were extremely low. In addition, the levels of *aqp1aa* mRNA in 2 yph gonads from different genotypes (male, female, pseudo-male, triploid male, and *DMRT1*-knock out) were determined. High levels of expression of *aqp1aa* were detected in the testis of males and pseudo-males, while the expression of *aqp1aa* was low in the ovary and lowest in the gonads of *DMRT1*-knock out fish and triploid male (Fig 2B).

The expression of *aqp1aa* at different stages of gonad development was also determined. In the larvae fish (34, 52, 80 days post-hatching, dph), the mRNA levels of *aqp1aa* were extremely low (Fig 2C). In the testis of ZZ males, the expression levels of *aqp1aa* continued to increase from 105 dph to 2 yph, until reaching a peak at 2 yph. Contrarily, in the ovary, no significant differences were observed before 2 yph, and the expression levels of *aqp1aa* were extremely low during the whole gonadal development.

### *In situ* hybridization

**Fig 3:**
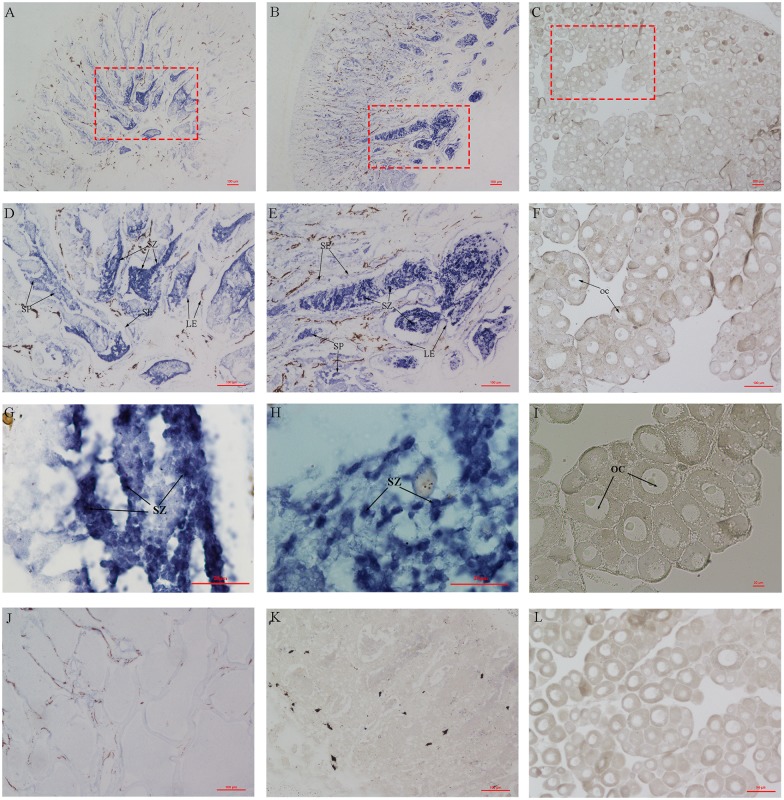
*In situ* localization of *aqp1aa* mRNA in 1 yph gonads of *C*. *semilaevis*. *In situ* hybridization of gonads using antisense (A-I) and sense (J-L) RNA probes of *aqp1aa* performed in half-smooth tongue sole. D (10x objective lens), E (10x), F (10x), G (100x), H (100x), and I (20x) are partial images zoomed in on particular areas of A, B, and C, respectively. (A, D, G, and J): testis of a ZZ male, (B, E, H, and K): testis of a ZW pseudo-male, (C, F, I, and L): ovary of a ZW female. SP: spermatid, SZ: spermatozoon, SE: Sertoli cells, LE: Leydig cells, OC: oocyte. Scale bar is shown in the figures.

To determine the distribution of the expression of *aqp1aa* in germ cells of 1 yph *C*. *semilaevis*, *in situ* hybridization assays were conducted. Extremely intense hybridization signals were observed in the testis of the males and pseudo-males (Fig 3D, 3E, 3G and 3H), especially in spermatozoa and spermatids, while no signals were detected in sertoli cells. In the ovary, no hybridization signal was detected in any type of cells (Fig 3F and 3I). Sense probes were used as a negative control (Fig 3J, 3K and 3L). These observations were also supported by the results of *in situ* hybridization assays in gonads at different developmental stages (210 dph and 2 yph), shown in S7 Fig. Besides, the histology of testis from 1 yph wild-type tongue sole was also analyzed to assist in distinguishing the cell type in testis (S8 Fig). There were a large amount of spermatozoa and spermatids (SZ and SP) in seminiferous cyst and some primary spermatocytes (PSC) and secondary spermatocytes (SSC) in the edge of seminiferous lobuli (SL). Secondary spermatocytes were larger than spermatozoa and spermatids, and primary spermatocytes were larger than secondary spermatocytes (S8 Fig).

## Discussion

Spermatogenesis is a complex process in which many functional genes are involved, and, among them, *aqp1aa* is a promising candidate gene. In this study, we cloned and characterized the full-length cDNA of *aqp1aa* in *C*. *semilaevis*. It encoded a 261 amino acids protein, which is a vital membrane intrinsic protein with the function of water channel [32, 58–59]. The protein consisted of 6 membrane-spanning α-helical domains forming a distinct pore, and contained the conserved NPA domains (Asn-Pro-Ala motifs) (S3 Table), which are specific to AQPs superfamily [58–61]. A phylogenetic analysis revealed that *aqp1aa* of *C*. *semilaevis* shared a high similarity with that of other teleosts [20], forming a well-supported cluster (pp = 1), especially with *O*. *niloticus*, *N*. *coriiceps*, *D*. *labrax*, and *O*. *latipes* with high posterior probability (pp = 0.86), while a low similarity was found with *aqp1aa* sequences from mammals, the latter forming another cluster with high clade support values (pp = 0.72) (Fig 1). Taken together, those results, supported by the alignment of amino acid sequences (S6 Fig), indicated that *aqp1aa* was highly conserved in teleosts. Different evolutionary statuses of *aqp1aa* suggest different functions of *aqp1aa* in teleosts and mammals. In mammals, in addition to being involved in cell migration and neural signal transduction [17, 34], AQPs also play a role in the early stages of spermatogenesis through the secretion of tubule liquid to achieve water homeostasis in the lumen, for sperm concentration transition and maturation [37]. In marine teleosts, *aqp1aa* mainly expressed in spermatozoa, especially located flagellum of the spermatozoa in the gilthead seabream (*Sparus aurata*) [38]. Besides, the *aqp1aa* is involved in the sperm motility activation by mediating the water efflux during hyperosmotic shock [38, 39, 43, 45]. However, the function of *aqp1aa* in *C*. *semilaevis* is still unknown.

To understand the expression pattern of *aqp1aa* in *C*. *semilaevis*, the mRNA expression levels of *aqp1aa* were analyzed using qPCR. *aqp1aa* was mainly expressed in spermatozoa and testis, suggesting its important role in male gonads, similarly to its function in rats and monkeys [62–63]. In addition, despite referring to low amounts, a significant difference in *aqp1aa* mRNA expression between males and females was detected in other tissues, especially in the brain (Fig 2A). The different functions of *aqp1aa* may exist in brain or other tissues, such as involving in the hypothalamic-pituitary-gonadal axis, but further study is needed to confirm this hypothesis. Furthermore, the mRNA expression levels of *aqp1aa* in both testis and ovaries were determined at different gonadal developmental stages. Low expression levels of *aqp1aa* were observed in the ovaries during the whole process, while high expression levels were found in testes from 105 dph to 2 yph, which is exactly the time when spermatogenesis occurs (Fig 2C) [2]. During that period, the testis differentiates with the completion of mitosis and the appearance of spermatocytes at 5 mph, the meiotic division of spermatocytes into spermatids at 8 mph, and the testis reaching maturity at 2 yph [2, 4]. Therefore, we speculate that *aqp1aa* plays a role in spermatogenesis during the process of sexual maturation.

The mRNA expression levels of *aqp1aa* in the gonads of different genotypes were also determined. High expression levels were detected in the testes of males and pseudo-males, while low expression levels were found in females and *DMRT1*-knock out fish, and no expression was detected in triploid males (Fig 2B). The genotype of a pseudo-male tongue sole is ZW; while the physiological sex is male, it can produce normal sperm similarly to ZZ males [2]. *DMRT1* is a vital gene that is involved in the male sex-determination of *C*. *semilaevis* [2]. In the gonads of *DMRT1*-knock out fish, no secondary spermatocytes, spermatids and sperms have been detected, and only spermatogonia and primary spermatocytes were found [5]. In addition, ZZZ triploid males cannot produce normal sperm due to the inhibition of testis development [4]. The above results show that the increase in the expression levels of *aqp1aa* mRNA is associated with the production of sperm. This result is additional evidence that indicates that *aqp1aa* may be involved in spermatogenesis.

The mRNA expression of *aqp1aa* was detected in testicular germ cells, including spermatozoon and spermatid, using *in situ* hybridization (Fig 3), which is identical to that in gilthead seabream [39, 45]. Furthermore, regarding its expression levels in gonads, *aqp1aa* was highly expressed in male and pseudo-male testis (Fig 3D, 3E, 3G and 3H), while it was hardly expressed in the ovary (Fig 3F and 3I), which is identical to the results obtained using qPCR. Based on these observations, we speculate that the *aqp1aa* in tongue sole may be involved in spermatogenesis. However, additional direct evidences are required to confirm this hypothesis.

In summary, we obtained the full-length cDNA of the *aqp1aa* gene in *C*. *semilaevis*. The mRNA expression data show that *aqp1aa* significantly expressed in testis during the process of sexual maturation. Besides, the results of *in situ* hybridization show that *aqp1aa* predominantly expressed in spermatozoon and spermatid of testis, indicating that *aqp1aa* may be involved in spermatogenesis in tongue sole. However, the further study is needed to confirm this speculation, such as the gene knock out, knock down, and immunofluorescence.

## Supporting information

S1 Fig The gonadal cell line of *C*.*semilaevis*. (**A**) The testis cell line at passage 12. (**B**) The ovary cell line at passage 12. Scale bar = 100 μm.
(TIF)

S2 Fig *β*-actin mRNA expression in *C*.*semilaevis* by semi-quantitative PCR and the validation of specificity for qPCR primer. (**A**) The expression of *β-* actin in various tissues of tongue sole. (**B**) The expression of *β*-actin in gonads of different genotypes. PM: ZW pseudo-male, M: ZZ male, F: ZW female, DMRT-knock out: DMRT1-knock out fish, TM: ZZZ triploid male. (**C**) The expression of *β*-actin at different developmental stages of the gonads. (**D**) PCR amplification of qPCR primer using cDNA and DNA as template.
(TIF)

S3 Fig The nucleotide sequences alignment of two *aqp1* paralogs from tongue sole. The specific region used to amplify the ISH probe is underlined.
(TIF)

S4 Fig The validation of nucleotide and predicted amino acid sequences of *aqp1aa* in *C*. *semilaevis*. The stop codon is indicated by the asterisk. The amphipathic channel is indicated by dots. The polyadenylation signal is marked by the shaded yellow box. Six TM helices are indicated by low dashes, and the asparagine—proline—alanine (NPA) motifs are marked by rounded rectangles. Three red amino acids stand for variations of amino acids induced by variations of nucleotide. The sequence of *aqp1aa* with genbank accession number HM013715 was published by Sun et al [40].
(TIF)

S5 Fig Schematic representation of *C*. *semilaevis aqp1aa* genomic structure. The capital letters (A-C) above the line represent the 3 introns, and the solid boxes (from 1 to 4) represent the 4 exons.
(TIF)

S6 Fig Multiple amino acid sequence alignment of *aqp1aa* protein from *C*. *semilaevis* and other vertebrates. Six transmembrane α-helices are underlined (from TM1 to TM6), and the asparagine—proline—alanine (NPA) motifs are boxed in blue.
(TIF)

S7 Fig *In situ* localization of *aqp1aa* mRNA in 210 dph and 2 yph gonads of *C*. *semilaevis*. Gonads *in situ* hybridization using antisense (A-D) and sense (E-H) RNA probe of *aqp1aa* performed in half-smooth tongue sole. A and B represent testis and ovary at 210 dph, respectively; C and D represent testis and ovary at 2 yph, respectively. Scale bars: 100 μm.
(TIF)

S8 Fig The histology of testis from 1 yph wild-type tongue sole. **A**, testis of control male. seminiferous lobuli (SL), seminiferous cyst (SC); **B**, larger magnification of frame area in A. primary spermatocytes (PSC), secondary spermatocytes (SSC), spermatid (SP), spermatozoon (SZ). Scale bar is shown in the figures.
(TIF)

S1 Table Primers and primer sequences used in this study. (DOC)

S2 Table List of species used in the phylogenetic analysis. (DOC)

S3 Table List of species used in the alignment of amino acid sequences. (DOC)

## References

[1] Shao CW, Wu PF, Wang XL, Tian YS, Chen SL. Comparison of chromosome preparation methods for the different developmental stages of the half-smooth tongue sole, (*Cynoglossus semilaevis*). Micron. 2010 1; 41(1): 47–50. 10.1016/j.micron.2009.08.002 19781952

[2] Chen S, Zhang G, Shao C, Huang Q, Liu G, Zhang P, et al Whole-genome sequence of a flatfish provides insights into ZW sex chromosome evolution and adaptation to a benthic lifestyle. Nat Genet. 2014 3; 46(3): 253–260. 10.1038/ng.2890 24487278

[3] Chen SL, Li WL, Ji XS, Xie MS, Xu Y, Deng H. Induction and identification of artificial triploid fry in *Cynoglossus semilaevis*. Journal of Fisheries of China. 2011 6; 35(6): 925–931.(In Chinese with English abstract)

[4] Pandian TJ, Koteeswaran R. Ploidy induction and sex control in fish. Hydrobiologia. 1998 1; 384(1): 167–243.

[5] Cui Z, Liu Y, Wang W, Wang Q, Zhang N, Lin F, et al Genome editing reveals dmrt1 as an essential male sex-determining gene in Chinese tongue sole (*Cynoglossus semilaevis*). Sci Rep. 2017 2; 7: 42213 10.1038/srep42213 28205594 PMC5311979

[6] Meng L, Zhu Y, Zhang N, Liu W, Liu Y, Shao C, et al Cloning and Characterization of *tesk1*, a Novel Spermatogenesis-Related Gene, in the Tongue Sole (*Cynoglossus semilaevis*). PLoS One. 2014 10; 9(10): e107922 10.1371/journal.pone.0107922 25271995 PMC4182740

[7] Zhang L, Liu W, Shao C, Zhang N, Li H, Liu K, et al Cloning, expression and methylation analysis of *piwil2* in half-smooth tongue sole (*Cynoglossus semilaevis*). Mar Genomics. 2014 12;18 Pt A: 45–54. 24794875 10.1016/j.margen.2014.04.004

[8] Li H, Xu W, Zhang N, Shao C, Zhu Y, Dong Z, et al Two Figla homologues have disparate functions during sex differentiation in half-smooth tongue sole (*Cynoglossus semilaevis*). Sci Rep. 2016 6; 6: 28219 10.1038/srep28219 27313147 PMC4911598

[9] Dong Z, Qi Q, Zhang N, Shao C, Zhang L, Wen H, et al Molecular characterization and expression analysis of *Patched 1* gene in the half-smooth tongue sole (*Cynoglossus semilaevis*). Acta Oceanol Sin. 2016 12; 35(6): 19–28.

[10] Xu W, Li H, Dong Z, Cui Z, Zhang N, Meng L, et al Ubiquitin ligase gene *neurl3* plays a role in spermatogenesis of half-smooth tongue sole (*Cynoglossus semilaevis*) by regulating testis protein ubiquitination. Gene. 2016 10; 592(1): 215–220. 10.1016/j.gene.2016.07.062 27480167

[11] Eddy EM. Chapter 1-The Spermatozoon. Knobil and Neill's Physiology of Reproduction. Elsevier Inc. 2006: 3–54

[12] Huang HF, He RH, Sun CC, Zhang Y, Meng QX, Ma YY. Function of aquaporins in female and male reproductive systems. Hum Reprod Update. 2006 Nov-Dec; 12(6): 785–795. 10.1093/humupd/dml035 16840793

[13] Rato L, Socorro S, Cavaco JE, Oliveira PF. Tubular fluid secretion in the seminiferous epithelium: ion transporters and aquaporins in Sertoli cells. J Membr Biol. 2010 7; 236(2): 215–224. 10.1007/s00232-010-9294-x 20697886

[14] Cooper TG, Yeung CH. Acquisition of volume regulatory response of sperm upon maturation in the epididymis and the role of the cytoplasmic droplet. Microsc Res Tech. 2003 5; 61(1): 28–38. 10.1002/jemt.10314 12672120

[15] Cosson J, Groison AL, Suquet M, Fauvel C, Dreanno C, Billard R. Studying sperm motility in marine fish: an overview on the state of the art. J Appl Ichthyol. 2008 2; 24(4): 460–486.

[16] Cosson J, Groison AL, Suquet M, Fauvel C, Dreanno C, Billard R. Marine fish spermatozoa: racing ephemeral swimmers. Reproduction. 2008 9; 136(3): 277–294. 10.1530/REP-07-0522 18524881

[17] Skowronski MT, Leska A, Robak A, Nielsen S. Immunolocalization of aquaporin-1, -5, and -7 in the avian testis and vas deferens. J Histochem Cytochem. 2009 10; 57(10): 915–922. 10.1369/jhc.2009.954057 19546471 PMC2746725

[18] Zardoya R. Phylogeny and evolution of the major intrinsic protein family. Biol Cell. 2005 6; 97(6): 397–414. 10.1042/BC20040134 15850454

[19] Finn RN, Cerdà J. Evolution and functional diversity of aquaporins. Biol Bull. 2015 8; 229(1): 6–23. 10.1086/BBLv229n1p6 26338866

[20] Finn RN, Chauvigné F, Hlidberg JB, Cutler CP, Cerdà J. The Lineage-Specific Evolution of Aquaporin Gene Clusters Facilitated Tetrapod Terrestrial Adaptation. PLoS One. 2014 11; 9(11): e113686 10.1371/journal.pone.0113686 25426855 PMC4245216

[21] Abascal F, Irisarri I, Zardoya R. Diversity and evolution of membrane intrinsic proteins. Biochim Biophys Acta. 2014 5; 1840(5): 1468–1481. 10.1016/j.bbagen.2013.12.001 24355433

[22] Ahmadpour D, Geijer C, Tamás MJ, Lindkvist-Petersson K, Hohmann S. Yeast reveals unexpected roles and regulatory features of aquaporins and aquaglyceroporins. Biochim Biophys Acta. 2014 5; 1840(5): 1482–1491. 10.1016/j.bbagen.2013.09.027 24076236

[23] Day RE, Kitchen P, Owen DS, Bland C, Marshall L, Conner AC, Bill RM, Conner MT. Human aquaporins: regulators of transcellular water flow. Biochim Biophys Acta. 2014 5; 1840(5): 1492–1506. 10.1016/j.bbagen.2013.09.033 24090884

[24] Ishibashi K, Tanaka Y, Morishita Y. The role of mammalian superaquaporins inside the cell. Biochim Biophys Acta. 2014 5; 1840(5): 1507–1512. 10.1016/j.bbagen.2013.10.039 24189537

[25] Li G, Santoni V, Maurel C. Plant aquaporins: roles in plant physiology. Biochim Biophys Acta. 2014 5; 1840(5): 1574–1582. 10.1016/j.bbagen.2013.11.004 24246957

[26] Mukhopadhyay R, Bhattacharjee H, Rosen BP. Aquaglyceroporins: generalized metalloid channels. Biochim Biophys Acta. 2014 5; 1840(5): 1583–1591. 10.1016/j.bbagen.2013.11.021 24291688 PMC3960311

[27] Song J, Mak E, Wu B, Beitz E. Parasite aquaporins: current developments in drug facilitation and resistance. Biochim Biophys Acta. 2014 5; 1840(5): 1566–1573. 10.1016/j.bbagen.2013.10.014 24140393

[28] Lee JK, Kozono D, Remis J, Kitagawa Y, Agre P, Stroud RM. Structural basis for conductance by the archaeal aquaporin AqpM at 1.68 Å. Proc Natl Acad Sci U S A. 2005 12; 102(52): 18932–18937. 10.1073/pnas.0509469102 16361443 PMC1323191

[29] Jiang J, Daniels BV, Fu D. Crystal structure of AqpZ tetramer reveals two distinct Arg-189 conformations associated with water permeation through the narrowest constriction of the water-conducting channel. J Biol Chem. 2006 1; 281(1): 454–460. 10.1074/jbc.M508926200 16239219

[30] Preston GM, Carroll TP, Guggino WB, Agre P. Appearance of water channels in Xenopus oocytes expressing red cell CHIP28 protein. Science. 1992 4; 256(5055): 385–387. 1373524 10.1126/science.256.5055.385

[31] Ren G, Reddy VS, Cheng A, Melnyk P, Mitra AK. Visualization of a water-selective pore by electron crystallography in vitreous ice. Proc Natl Acad Sci USA. 2001 2; 98(4): 1398–1403. 10.1073/pnas.98.4.1398 11171962 PMC29268

[32] Sui H, Han BG, Lee JK, Walian P, Jap BK. Structural basis of water-specific transport through the AQP1 water channel. Nature. 2001 12; 414(6866): 872–878. 10.1038/414872a 11780053

[33] Verkman AS. More than just water channels: unexpected cellular roles of aquaporins. J Cell Sci. 2005 8;118(15): 3225–3232. 16079275 10.1242/jcs.02519

[34] Zhang H, Verkman AS. Aquaporin-1 water permeability as a novel determinant of axonal regeneration in dorsal root ganglion neurons. Exp Neurol. 2015 3; 265: 152–159. 10.1016/j.expneurol.2015.01.002 25585012 PMC4346453

[35] Brown D, Verbavatz JM, Valenti G, Lui B, Sabolic I. Localization of the CHIP28 water channel in reabsorptive segments of the rat male reproductive tract. Eur J Cell Biol. 1993 8; 61(2): 264–273. 8223717

[36] Fisher JS, Turner KJ, Fraser HM, Saunders PTK, Brown D, Sharpe RM. Immunoexpression of Aquaporin-1 in the efferent ducts of the rat and marmoset monkey during development, its modulation by estrogens, and its possible role in fluid resorption. Endocrinology. 1998 9; 139(9): 3935–3945. 10.1210/endo.139.9.6213 9724049

[37] Dacheux JL, Castella S, Gatti JL, Dacheux F. Epididymal cell secretory activities and the role of proteins in boar sperm maturation. Theriogenology. 2005 1; 63(2): 319–341. 10.1016/j.theriogenology.2004.09.015 15626402

[38] Zilli L, Schiavone R, Chauvigné F, Cerdà J, Storelli C, Vilella S. Evidence for the involvement of aquaporins in sperm motility activation of the teleost gilthead sea bream (*Sparus aurata*). Biol Reprod. 2009 11; 81(5): 880–888. 10.1095/biolreprod.109.077933 19571262

[39] Boj M, Chauvigné F, Cerdà J. Coordinated Action of Aquaporins Regulates Sperm Motility in a Marine Teleost. Biol Reprod. 2015 8; 93(2): 40 10.1095/biolreprod.115.131524 26134868

[40] Sun Y, Zhang Q, Qi J, Yu Y, Li S, Li C, et al Cloning, Expression and Analysis of Two Aquaporin1 Paralogous Genes in *Cynoglossus semilaevis*. 2009 6; 55(3): 335–339. (In Chinese with English abstract)

[41] Fabra M, Raldúa D, Power DM, Deen PM, Cerdà J. Marine Fish Egg Hydration Is Aquaporin-Mediated. Science. 2005 1; 307(5709): 545 10.1126/science.1106305 15681377

[42] An KW, Kim NN, Choi CY. Cloning and expression of aquaporin 1 and arginine vasotocin receptor mRNA from the black porgy, *Acanthopagrus schlegeli*: effect of freshwater acclimation. Fish Physiol Biochem. 2008 6; 34(2): 185–194. 10.1007/s10695-007-9175-0 18649036

[43] Chauvigné F, Boj M, Vilella S, Finn RN, Cerdà J. Subcellular localization of selectively permeable aquaporins in the male germ line of a marine teleost reveals spatial redistribution in activated spermatozoa. Biol Reprod. 2013 8; 89(2): 37 10.1095/biolreprod.113.110783 23782838

[44] Cerdà J, Finn RN. Piscine aquaporins: an overview of recent advances. J Exp Zool A Ecol Genet Physiol. 2010 12; 313(10): 623–650. 10.1002/jez.634 20717996

[45] Boj M, Chauvigné F, Cerdà J. Aquaporin Biology of Spermatogenesis and Sperm Physiology in Mammals and Teleosts. Biol Bull. 2015 8; 229(1): 93–108. 10.1086/BBLv229n1p93 26338872

[46] Zapater C, Chauvigné F, Norberg B, Finn RN, Cerdà J. Dual neofunctionalization of a rapidly evolving aquaporin-1 paralog resulted in constrained and relaxed traits controlling channel function during meiosis resumption in teleosts. Mol Biol Evol. 2011 11; 28(11): 3151–3169. 10.1093/molbev/msr146 21653921

[47] Zapater C, Chauvigné F, Scott AP, Gómez A, Katsiadaki I, Cerdà J. Piscine follicle-stimulating hormone triggers progestin production in gilthead seabream primary ovarian follicles. Biol Reprod. 2012 11; 87(5): 111 10.1095/biolreprod.112.102533 22976280

[48] Zapater C, Chauvigné F, Tingaud-Sequeira A, Finn RN, Cerdà J. Primary oocyte transcriptional activation of aqp1ab by the nuclear progestin receptor determines the pelagic egg phenotype of marine teleosts. Dev Biol. 2013 5; 377(2): 345–362. 10.1016/j.ydbio.2013.03.001 23499660

[49] Zilli L, Beirão J, Schiavone R, Herraez MP, Cabrita E, Storelli C, et al Aquaporin inhibition changes protein phosphorylation pattern following sperm motility activation in fish. 2011 9; 76(4): 737–744. 10.1016/j.theriogenology.2011.04.006 21620454

[50] Chen SL, Ji XS, Shao CW, Li WL, Yang JF, Liang Z, et al Induction of Mitogynogenetic Diploids and Identification of WW Super-female Using Sex-Specific SSR Markers in Half-Smooth Tongue Sole (*Cynoglossus semilaevis*). Mar Biotechnol (NY). 2012 2; 14(1): 120–128. 21735350 10.1007/s10126-011-9395-2

[51] Sun A, Wang TZ, Wang N, Liu XF, Sha ZX, Chen SL. Establishment and characterization of an ovarian cell line from half-smooth tongue sole *Cynoglossus semilaevis*. J Fish Biol. 2015 1; 86(1): 46–59. 10.1111/jfb.12535 25359438

[52] Zhang B, Wang X, Sha Z, Yang C, Liu S, Wang N, et al Establishment and characterization of a testicular cell line from the half-smooth tongue sole, *Cynoglossus semilaevis*. Int J Biol Sci. 2011 4; 7(4): 452–459. 21547062 10.7150/ijbs.7.452 PMC3088287

[53] Källberg M, Wang H, Wang S, Peng J, Wang Z, Lu H, et al Template-based protein structure modeling using the RaptorX web server. Nat Protoc. 2012 7; 7(8): 1511–1522. 10.1038/nprot.2012.085 22814390 PMC4730388

[54] Lanfear R, Calcott B, Ho SYW, Guindon S. PartitionFinder: combined selection of partitioning schemes and substitution models for phylogenetic analyses. Mol Biol Evol. 2012 6; 29(6):1695–1701. 10.1093/molbev/mss020 22319168

[55] Ronquist F & Huelsenbeck JP. MrBayes 3: Bayesian phylogenetic inference under mixed models. Bioinformatics 2003 8; 19 (12): 1572–1574. 12912839 10.1093/bioinformatics/btg180

[56] Li Z, Yang L, Wang J, Shi W, Pawar RA, Liu Y, et al beta-Actin is a useful internal control for tissue-specific gene expression studies using quantitative real-time PCR in the half-smooth tongue sole Cynoglossus semilaevis challenged with LPS or Vibrio anguillarum. Fish Shellfish Immunol. 2010 7; 29(1): 89–93. 10.1016/j.fsi.2010.02.021 20227507

[57] Livak KJ, Schmittgen TD. Analysis of relative gene expression data using real-time quantitative PCR and the 2(-Delta Delta C(T)) Method. Methods. 2001 12; 25(4): 402–408. 10.1006/meth.2001.1262 11846609

[58] Verkman AS, Mitra AK. 2000. Structure and function of aquaporin water channels. Am J Physiol Renal Physiol. 2000 1; 278(1): F13–28. 10644652 10.1152/ajprenal.2000.278.1.F13

[59] Yool AJ, Campbell EM. Structure, function and translational relevance of aquaporin dual water and ion channels. Mol Aspects Med. 2012 Oct-Dec; 33(5–6): 553–561. 10.1016/j.mam.2012.02.001 22342689 PMC3419283

[60] King LS, Agre P. Pathophysiology of the Aquaporin Water Channels. Annu Rev Physiol. 1996 3; 58: 619–648. 10.1146/annurev.ph.58.030196.003155 8815812

[61] Wintour E.M., 1997. Water channels and urea transporters. Clin Exp Pharmacol Physiol. 1997 1; 24: 1–9. 9043798 10.1111/j.1440-1681.1997.tb01775.x

[62] Brown D, Verbavatz JM, Valenti G, Lui B, Sabolic I. Localization of the CHIP28 water channel in reabsorptive segments of the rat male reproductive tract. Eur J Cell Biol. 1993 8; 61(2): 264–273. 8223717

[63] Fisher JS, Turner KJ, Fraser HM, Saunders PTK, Brown D, Sharpe RM. Immunoexpression of Aquaporin-1 in the efferent ducts of the rat and marmoset monkey during development, its modulation by estrogens, and its possible role in fluid resorption. Endocrinology. 1998 9; 139(9): 3935–3945. 10.1210/endo.139.9.6213 9724049

