# Supplementary figures and images for: Molecular cloning and expression analysis of the aqp1aa gene in half-smooth tongue sole (Cynoglossus semilaevis)
Source: PLoS One. 2017 Apr 5;12(4):e0175033. doi: 10.1371/journal.pone.0175033 (PMC5381947; doi:10.1371/journal.pone.0175033)

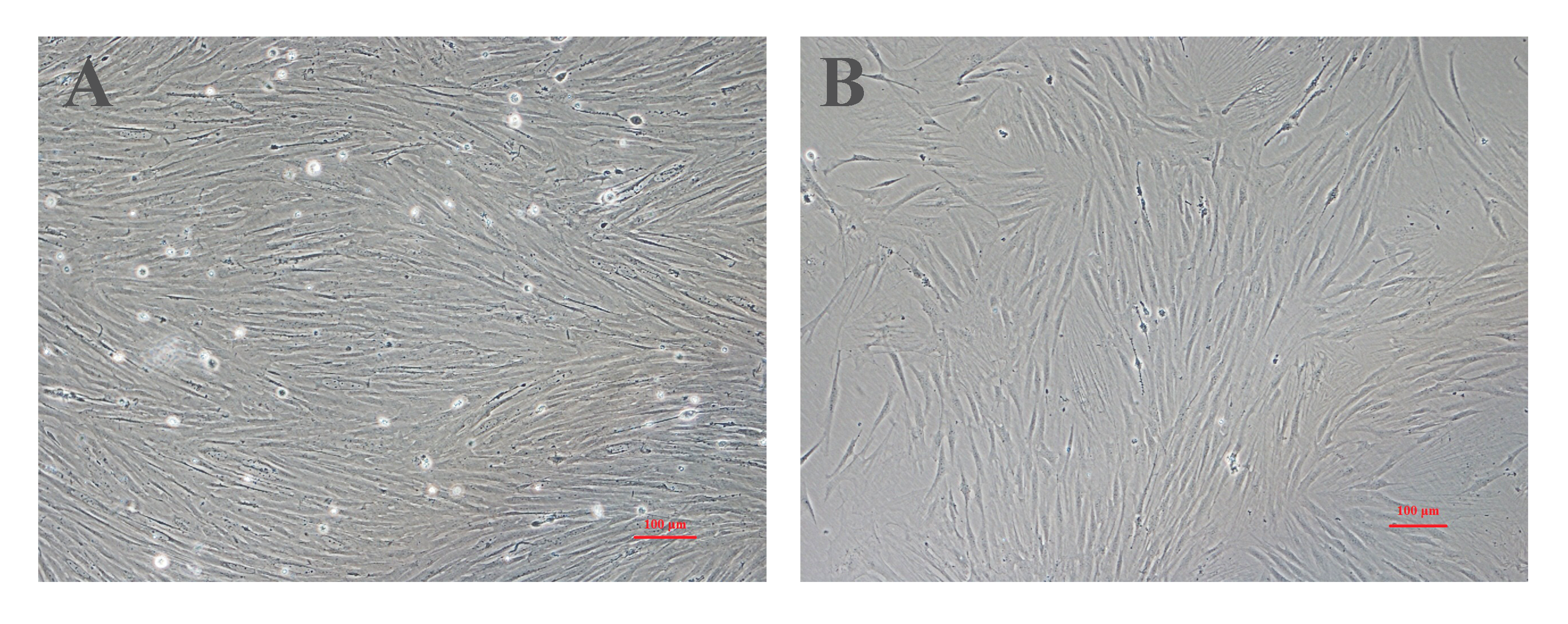

Supplement: S1 Fig — (A) The testis cell line at passage 12. (B) The ovary cell line at passage 12. Scale bar = 100 μm. (TIF) [file pone.0175033.s001.tif]

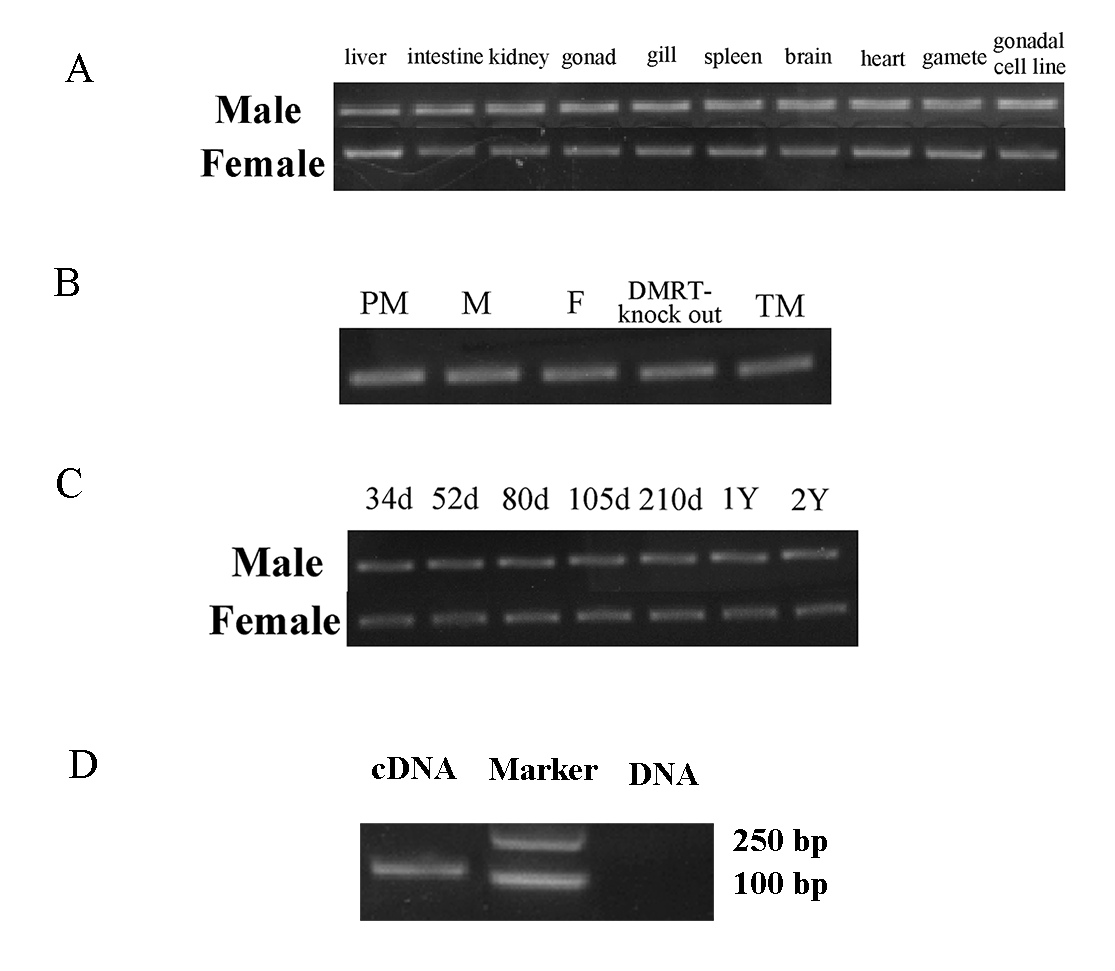

Supplement: S2 Fig — (A) The expression of β- actin in various tissues of tongue sole. (B) The expression of β-actin in gonads of different genotypes. PM: ZW pseudo-male, M: ZZ male, F: ZW female, DMRT-knock out: DMRT1-knock out fish, TM: ZZZ triploid male. (C) The expression of β-actin at different developmental stages of the gonads. (D) PCR amplification of qPCR primer using cDNA and DNA as template. (TIF) [file pone.0175033.s002.tif]

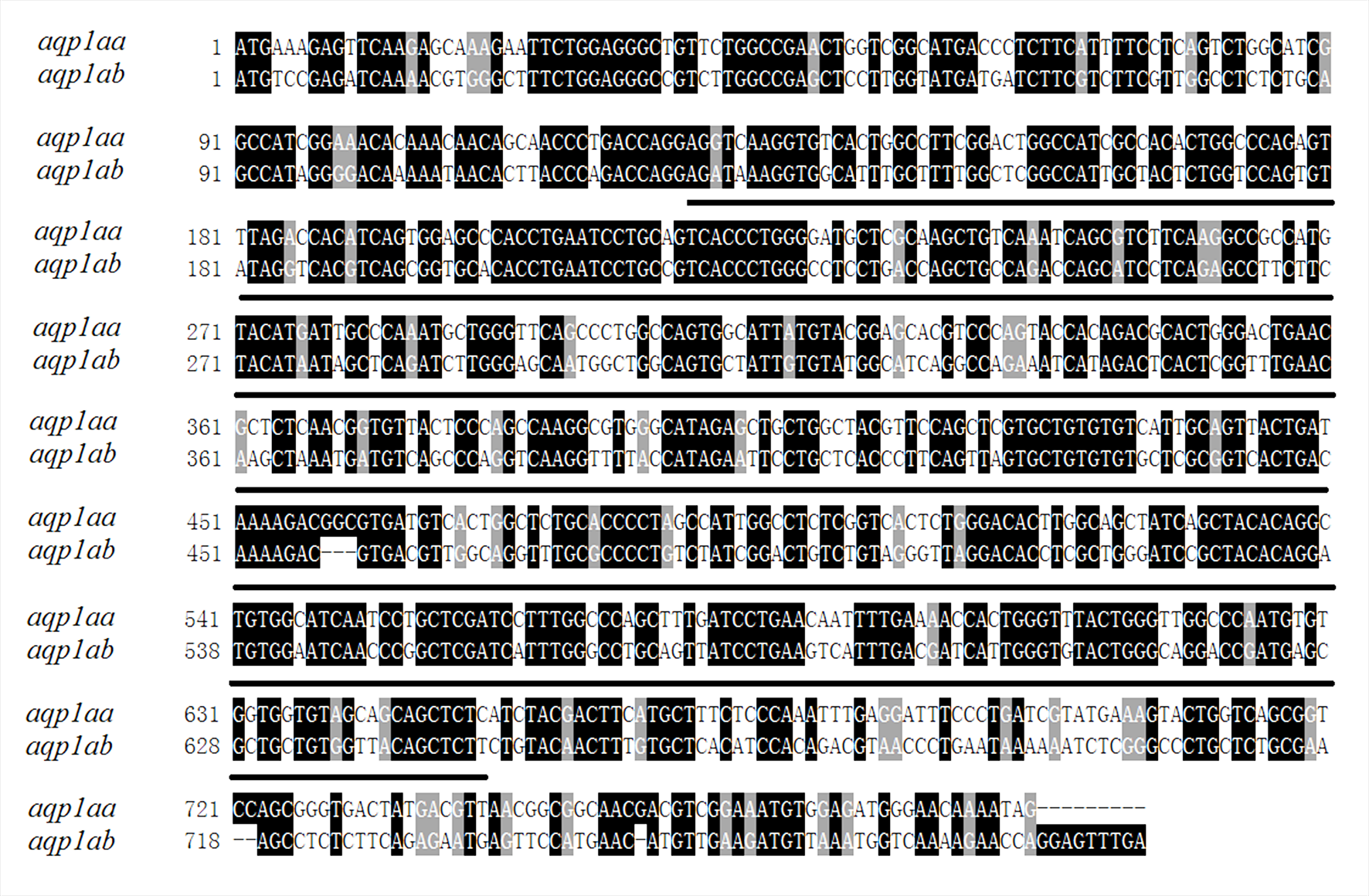

Supplement: S3 Fig — The specific region used to amplify the ISH probe is underlined. (TIF) [file pone.0175033.s003.tif]

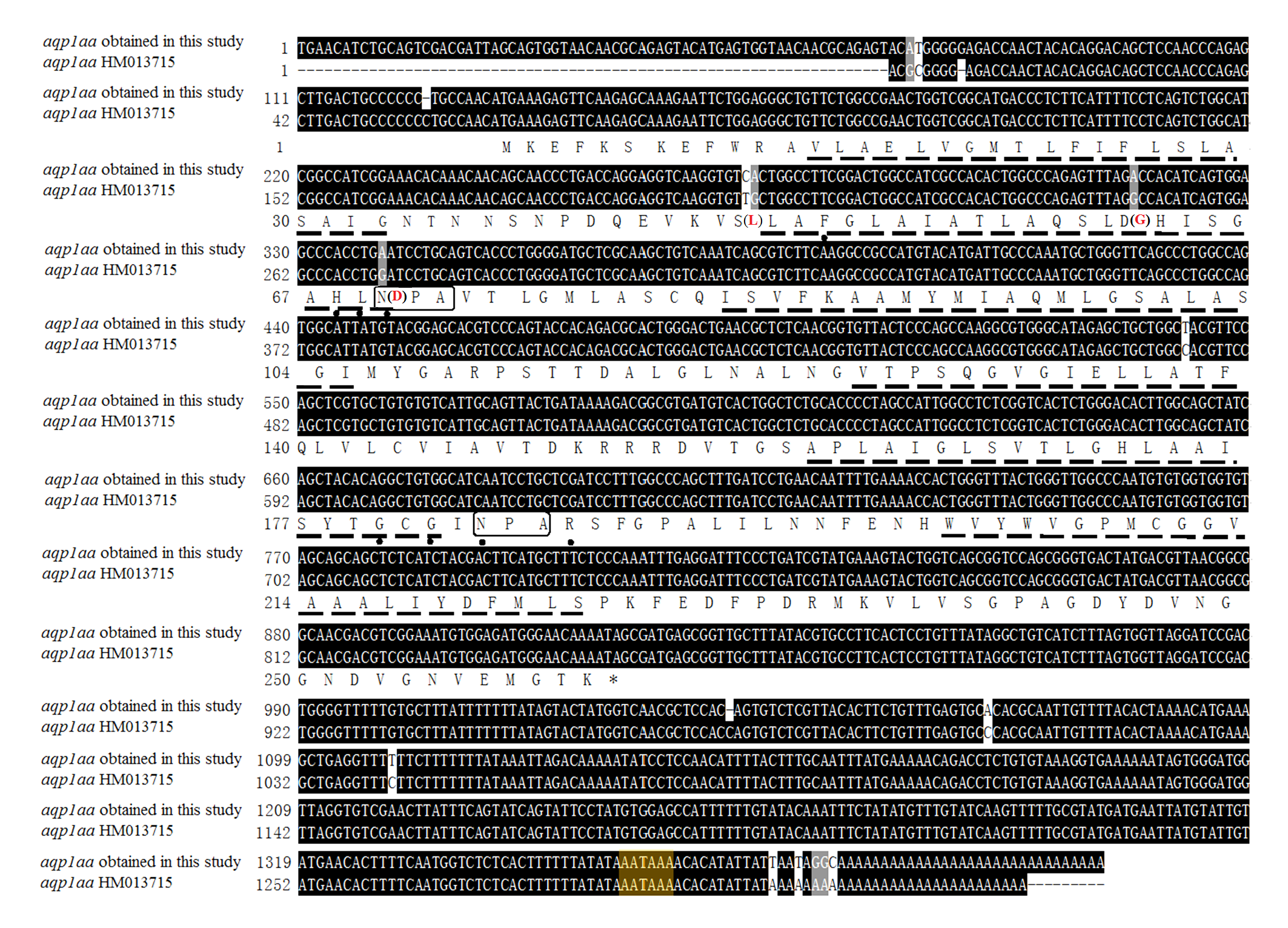

Supplement: S4 Fig — The stop codon is indicated by the asterisk. The amphipathic channel is indicated by dots. The polyadenylation signal is marked by the shaded yellow box. Six TM helices are indicated by low dashes, and the asparagine—proline—alanine (NPA) motifs are marked by rounded rectangles. Three red amino acids stand for variations of amino acids induced by variations of nucleotide. The sequence of aqp1aa with genbank accession number HM013715 was published by Sun et al [40]. (TIF) [file pone.0175033.s004.tif]

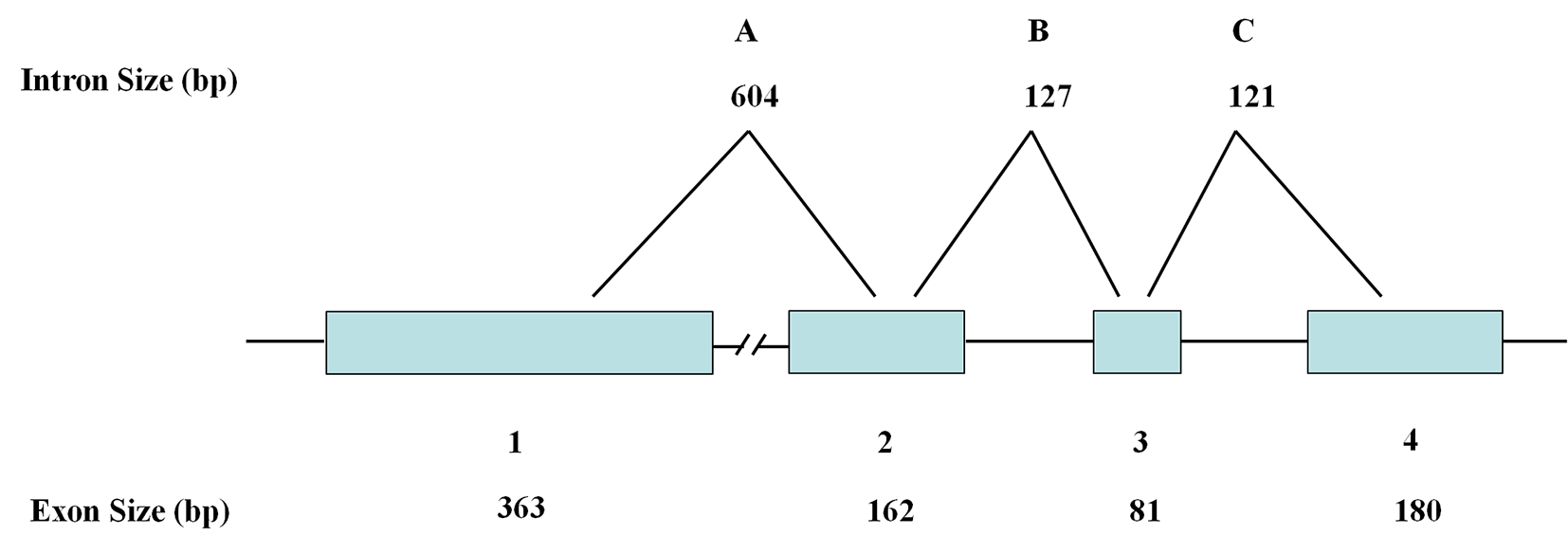

Supplement: S5 Fig — The capital letters (A-C) above the line represent the 3 introns, and the solid boxes (from 1 to 4) represent the 4 exons. (TIF) [file pone.0175033.s005.tif]

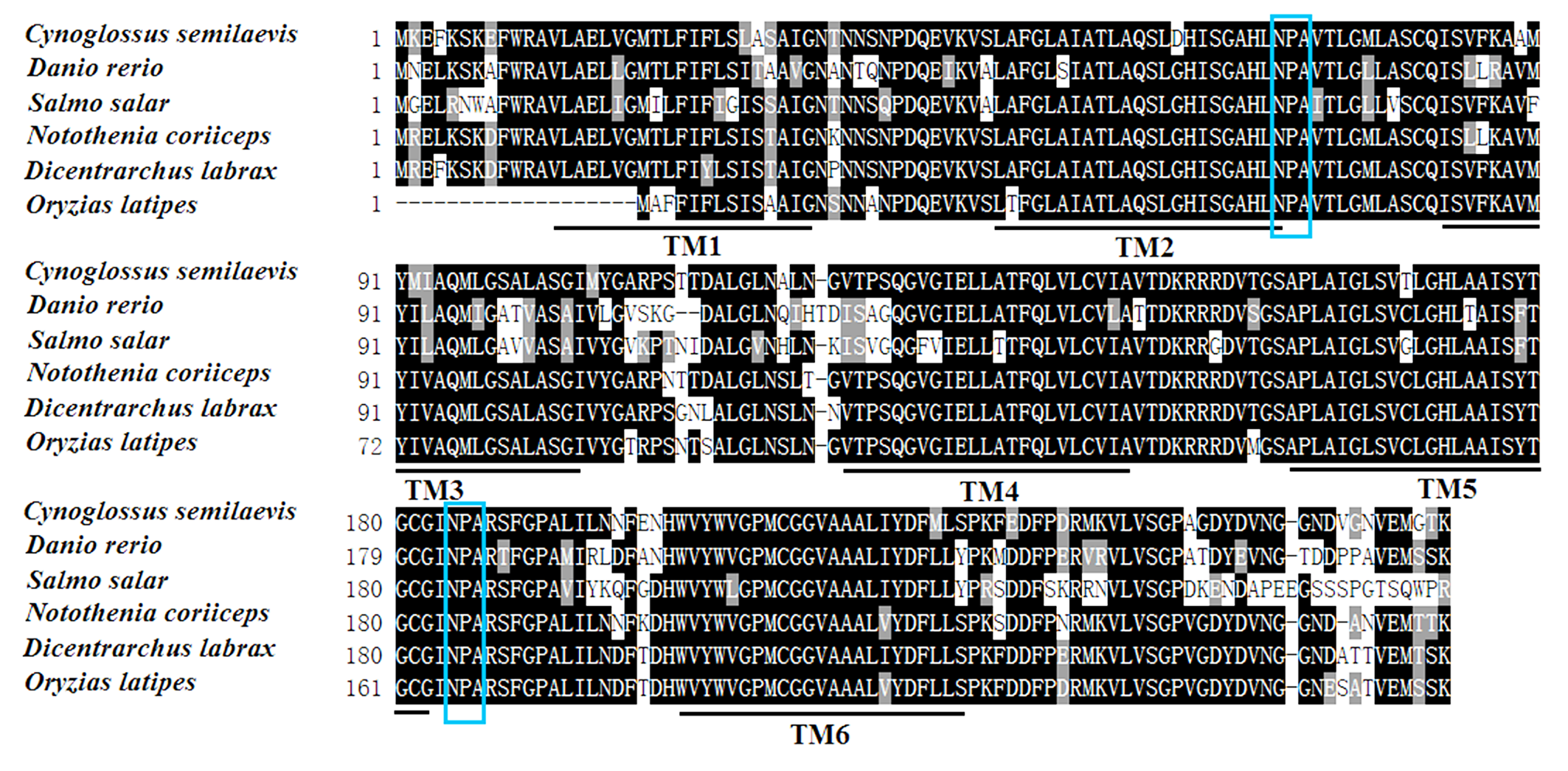

Supplement: S6 Fig — Six transmembrane α-helices are underlined (from TM1 to TM6), and the asparagine—proline—alanine (NPA) motifs are boxed in blue. (TIF) [file pone.0175033.s006.tif]

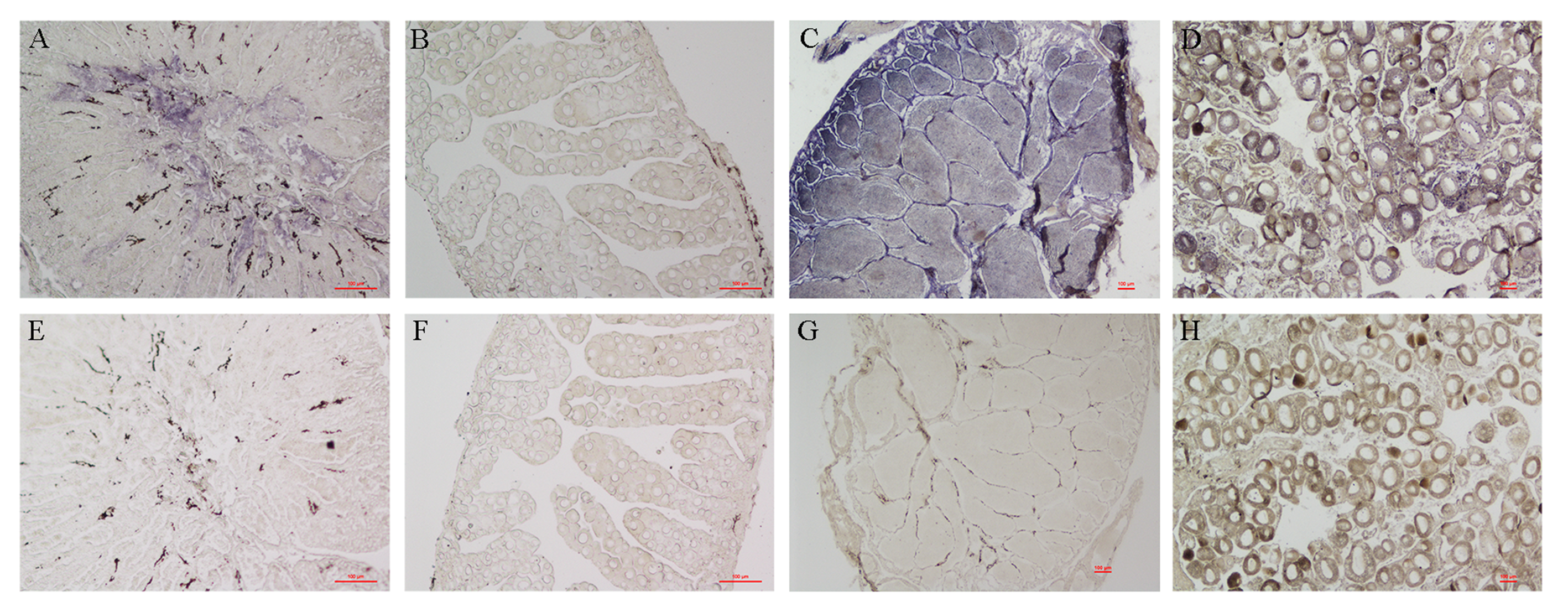

Supplement: S7 Fig — Gonads in situ hybridization using antisense (A-D) and sense (E-H) RNA probe of aqp1aa performed in half-smooth tongue sole. A and B represent testis and ovary at 210 dph, respectively; C and D represent testis and ovary at 2 yph, respectively. Scale bars: 100 μm. (TIF) [file pone.0175033.s007.tif]

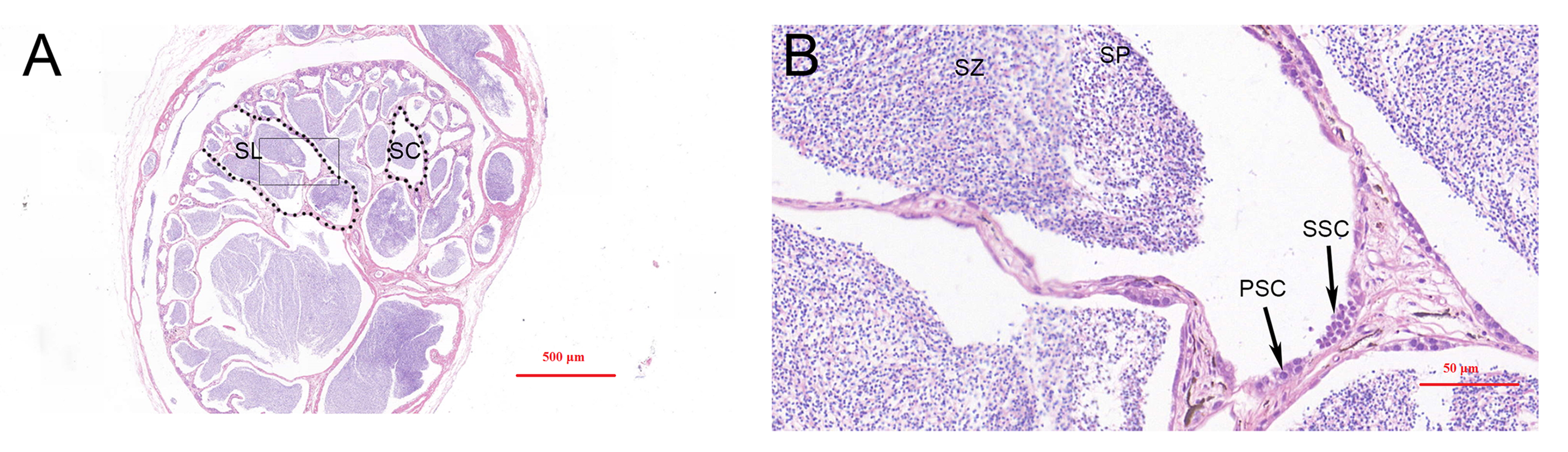

Supplement: S8 Fig — A, testis of control male. seminiferous lobuli (SL), seminiferous cyst (SC); B, larger magnification of frame area in A. primary spermatocytes (PSC), secondary spermatocytes (SSC), spermatid (SP), spermatozoon (SZ). Scale bar is shown in the figures. (TIF) [file pone.0175033.s008.tif]
