# Supplementary material for: Molecular cloning and expression analysis of the aqp1aa gene in half-smooth tongue sole (Cynoglossus semilaevis)
Source: PLoS One. 2017 Apr 5;12(4):e0175033. doi: 10.1371/journal.pone.0175033 (PMC5381947; doi:10.1371/journal.pone.0175033)
Supplement: S1 Table — (DOC) [file pone.0175033.s009.doc]

**S1 Table Primers and their sequences in this study**.

| primer | Sequence (5′-3′) | Purpose | Product size |
| --- | --- | --- | --- |
| AQP1-F | CTGAGACCAACTACACAGG | Partial fragment cloning | 483bp |
| AQP1-R | CACGAGCTGGAACGTAGCC | Partial fragment cloning | 483bp |
| AQP1-GSP5′ | TTGGCTGGGAGTAACACCGTTGAGAGC | 5′RACE |  |
| AQP1-NGSP5′ | GACGTGCTCCGTACATAATGC | 5′RACE |  |
| AQP1-GSP3′ | GACCAACTACACAGGACAGCTCCAACC | 3′RACE |  |
| AQP1-NGSP3′ | AATTCTGGAGGGCTGTTCTGG | 3′RACE |  |
| Upm-long | CTAATACGACTCACTATAGGGCAAGCAGTGGTATCAACGCAGAGT | RACE |  |
| Upm-short | CTAATACGACTCACTATAGGGC | RACE |  |
| NUP | AAGCAGTGGTAACAACGCAGAGT | RACE |  |
| AQP1-RT-F | TGGGACTGAACGCTCTCAAC | RT-qPCR | 122bp |
| AQP1-RT-F | AGTGACATCACGCCGTCTTT | RT-qPCR | 122bp |
| actinF | GCTGTGCTGTCCCTGTA | Internal reference | 185bp |
| actinR | GAGTAGCCACGCTCTGTC | Internal reference | 185bp |
| AQP1-ISH-F | AGGTCAAGGTGTCACTGGC | ISH probe | 522bp |
| AQP1-ISH-R | GAGCTGCTGCTACACCACC | ISH probe | 522bp |
