# Supplementary material for: Molecular cloning and expression analysis of the aqp1aa gene in half-smooth tongue sole (Cynoglossus semilaevis)
Source: PLoS One. 2017 Apr 5;12(4):e0175033. doi: 10.1371/journal.pone.0175033 (PMC5381947; doi:10.1371/journal.pone.0175033)
Supplement: S2 Table — (DOC) [file pone.0175033.s010.doc]

**S2 Table** **List of species used in phylogenetic analysis**

| No | Species name | Category | Accession ID | source |
| --- | --- | --- | --- | --- |
| 1 | *Cynoglossus semilaevis* | Teleostes | KX904930 | Obtained in this study |
| 2 | *Stegastes partitus* |  | XM_008282895 | GenBank |
| 3 | *Diplodus sargus* |  | JN210582 |  |
| 4 | *Oreochromis niloticus* |  | XM_003438085 |  |
| 5 | *Acanthopagrus schlegelii* |  | EF451961 |  |
| 6 | *Notothenia coriiceps* |  | XM_010767642 |  |
| 7 | *Dicentrarchus labrax* |  | DQ924529 |  |
| 8 | *Sparus aurata* |  | AY626939 |  |
| 9 | *Cyprinodon variegatus* |  | XM_015369857 |  |
| 10 | *Rhabdosargus sarba* |  | JF803845 |  |
| 11 | *Poecilia latipinna* |  | XM_015034612 |  |
| 12 | *Xiphophorus maculatus* |  | XM_005809446 |  |
| 13 | *Poecilia mexicana* |  | XM_014980920 |  |
| 14 | *Oryzias latipes* |  | XM_011487012 |  |
| 15 | *Fundulus heteroclitus* |  | NM_001309974 |  |
| 16 | *Danio-rerio* |  | AY626937 |  |
| 17 | *Salmo salar* |  | NM_001140000 |  |
| 18 | *Mus musculus* | Mammals | NM_007472 |  |
| 19 | *Bos taurus* |  | NM_174702 |  |
| 20 | *Sus scrofa* |  | NM_214454 |  |
| 21 | *Tursiops truncatus* |  | NM_001287462 |  |
| 22 | *Ovis aries* |  | NM_001009194 |  |
| 23 | *Homo sapiens* |  | AB451275 |  |
| 24 | *Ciona intestinalis* | Invertebrates | XM_002128914 |  |
