# Supplementary material for: Molecular cloning and expression analysis of the aqp1aa gene in half-smooth tongue sole (Cynoglossus semilaevis)
Source: PLoS One. 2017 Apr 5;12(4):e0175033. doi: 10.1371/journal.pone.0175033 (PMC5381947; doi:10.1371/journal.pone.0175033)
Supplement: S3 Table — (DOC) [file pone.0175033.s011.doc]

**S3 Table List of species used in alignment of amino acid sequences**

| No | Species name | Accession ID |
| --- | --- | --- |
| 1 | *Cynoglossus semilaevis* | predicted |
| 2 | *Danio rerio* | AAV34608 |
| 3 | *Salmo salar* | NP_001133472 |
| 4 | *Notothenia coriiceps* | XP_010765944 |
| 5 | *Dicentrarchus labrax* | ABI95464 |
| 6 | *Oryzias latipes* | XP_011485314 |
